# Supplementary material for: Multi‐dataset identification of innovative feature genes and molecular mechanisms in keratoconus
Source: J Cell Mol Med. 2024 Sep 19;28(18):e70079. doi: 10.1111/jcmm.70079 (PMC11412914; doi:10.1111/jcmm.70079)
Supplement: Supplementary file 2 — Material S2. [file JCMM-28-e70079-s002.zip › 7.GSEA analysis/4.MYRF/KEGG_GRAFT_VERSUS_HOST_DISEASE.html]

Details for gene set KEGG\_GRAFT\_VERSUS\_HOST\_DISEASE[GSEA]

|  || Dataset | MYRF.group.cls#h\_versus\_l |
| Phenotype | group.cls#h\_versus\_l |
| Upregulated in class | l |
| GeneSet | KEGG\_GRAFT\_VERSUS\_HOST\_DISEASE |
| Enrichment Score (ES) | -0.6234075 |
| Normalized Enrichment Score (NES) | -1.5769283 |
| Nominal p-value | 0.00990099 |
| FDR q-value | 0.22127953 |
| FWER p-Value | 0.65 |
Table: GSEA Results Summary

  

Fig 1: Enrichment plot: KEGG\_GRAFT\_VERSUS\_HOST\_DISEASE      
 Profile of the Running ES Score & Positions of GeneSet Members on the Rank Ordered List

  

| SYMBOL | TITLE | RANK IN GENE LIST | RANK METRIC SCORE | RUNNING ES | CORE ENRICHMENT || 1 | CD86 | na | 355 | 0.626 | 0.0621 | No |
| 2 | HLA-DOA | na | 8167 | 0.134 | -0.1475 | No |
| 3 | KIR3DL2 | na | 9964 | 0.096 | -0.1881 | No |
| 4 | KLRC1 | na | 10155 | 0.093 | -0.1828 | No |
| 5 | CD28 | na | 12283 | 0.059 | -0.2373 | No |
| 6 | HLA-B | na | 12962 | 0.051 | -0.2509 | No |
| 7 | HLA-F | na | 16261 | 0.015 | -0.3443 | No |
| 8 | HLA-E | na | 16263 | 0.015 | -0.3426 | No |
| 9 | FASLG | na | 16299 | 0.015 | -0.3419 | No |
| 10 | KIR2DL2 | na | 17132 | 0.008 | -0.3650 | No |
| 11 | KIR2DL5A | na | 17527 | 0.004 | -0.3759 | No |
| 12 | HLA-C | na | 17654 | 0.003 | -0.3792 | No |
| 13 | HLA-A | na | 20084 | -0.023 | -0.4466 | No |
| 14 | HLA-DRB5 | na | 20183 | -0.024 | -0.4466 | No |
| 15 | HLA-G | na | 20210 | -0.024 | -0.4446 | No |
| 16 | GZMB | na | 22901 | -0.058 | -0.5154 | No |
| 17 | KLRD1 | na | 23082 | -0.061 | -0.5136 | No |
| 18 | HLA-DRB3 | na | 23156 | -0.062 | -0.5085 | No |
| 19 | HLA-DQB1 | na | 24038 | -0.076 | -0.5250 | No |
| 20 | HLA-DRB1 | na | 24046 | -0.077 | -0.5164 | No |
| 21 | HLA-DOB | na | 24105 | -0.078 | -0.5091 | No |
| 22 | CD80 | na | 26452 | -0.122 | -0.5626 | No |
| 23 | HLA-DMA | na | 27498 | -0.148 | -0.5756 | No |
| 24 | FAS | na | 28092 | -0.163 | -0.5739 | No |
| 25 | KIR3DL1 | na | 29811 | -0.217 | -0.5983 | Yes |
| 26 | HLA-DRB4 | na | 30247 | -0.232 | -0.5840 | Yes |
| 27 | PRF1 | na | 31281 | -0.279 | -0.5815 | Yes |
| 28 | HLA-DPA1 | na | 32053 | -0.320 | -0.5667 | Yes |
| 29 | IL1A | na | 32896 | -0.380 | -0.5471 | Yes |
| 30 | HLA-DRA | na | 33031 | -0.390 | -0.5059 | Yes |
| 31 | IFNG | na | 33150 | -0.402 | -0.4628 | Yes |
| 32 | HLA-DMB | na | 33256 | -0.414 | -0.4180 | Yes |
| 33 | IL6 | na | 33353 | -0.425 | -0.3717 | Yes |
| 34 | IL2 | na | 33402 | -0.431 | -0.3232 | Yes |
| 35 | HLA-DQA2 | na | 34217 | -0.576 | -0.2800 | Yes |
| 36 | IL1B | na | 34248 | -0.586 | -0.2132 | Yes |
| 37 | TNF | na | 34328 | -0.609 | -0.1451 | Yes |
| 38 | HLA-DPB1 | na | 34331 | -0.609 | -0.0747 | Yes |
| 39 | HLA-DQA1 | na | 34602 | -0.745 | 0.0036 | Yes |
Table: GSEA details [plain text format]

  

Fig 2: KEGG\_GRAFT\_VERSUS\_HOST\_DISEASE      
 Blue-Pink O' Gram in the Space of the Analyzed GeneSet

  

Fig 3: KEGG\_GRAFT\_VERSUS\_HOST\_DISEASE: Random ES distribution      
 Gene set null distribution of ES for **KEGG\_GRAFT\_VERSUS\_HOST\_DISEASE**
